# Developing a New Wireless Sensor Network Platform and Its Application in Precision Agriculture

**DOI:** 10.3390/s110101192

**Published:** 2011-01-20

**Authors:** Raúl Aquino-Santos, Apolinar González-Potes, Arthur Edwards-Block, Raúl Alejandro Virgen-Ortiz

**Affiliations:** 1 Faculty of Telematics, University of Colima, Av. University 333, C. P. 28040, Colima, Col., Mexico; E-Mail: arted@ucol.mx; 2 Faculty of Electrical and Mechanical Engineering, University of Colima, Coquimatlán-Colima, Km.2, Coquimatlán, Col., Mexico; E-Mail: apogon@ucol.mx; 3 SITELDI Solutions., Canarios 111, C. P. 28017, Colima, Col., Mexico; E-Mail: raul.virgen@siteldisolutions.com

**Keywords:** sensor networks, routing algorithm, technological platform, precision agriculture

## Abstract

Wireless sensor networks are gaining greater attention from the research community and industrial professionals because these small pieces of “smart dust” offer great advantages due to their small size, low power consumption, easy integration and support for “green” applications. Green applications are considered a hot topic in intelligent environments, ubiquitous and pervasive computing. This work evaluates a new wireless sensor network platform and its application in precision agriculture, including its embedded operating system and its routing algorithm. To validate the technological platform and the embedded operating system, two different routing strategies were compared: hierarchical and flat. Both of these routing algorithms were tested in a small-scale network applied to a watermelon field. However, we strongly believe that this technological platform can be also applied to precision agriculture because it incorporates a modified version of LORA-CBF, a wireless location-based routing algorithm that uses cluster-based flooding. Cluster-based flooding addresses the scalability concerns of wireless sensor networks, while the modified LORA-CBF routing algorithm includes a metric to monitor residual battery energy. Furthermore, results show that the modified version of LORA-CBF functions well with both the flat and hierarchical algorithms, although it functions better with the flat algorithm in a small-scale agricultural network.

## 1. Introduction

Agricultural production represents a strategic sector of any national economy. This is particularly true in Latin American countries, where traditionally a large percentage of the population is rural and depends on traditional agricultural production to live. Efficiently monitoring crops is critical because it significantly increases production, rationalizes the use of water and other consumables, and produces value-added crops [1]. However, crop monitoring can sometimes present technological difficulties, thus increasing operational costs and maintenance. The cost and maintenance of complex systems often exceeds what smaller farmers can invest. Smaller producers in developing countries cannot exploit the benefits of a scale economy. Unfortunately, because smaller, traditional producers often cannot compete, they abandon their fields seeking improved economic opportunities in the cities. In Latin America countries, approximately 40% of the population has migrated from rural to urban settings either in their own countries or abroad [2]. This migratory trend has resulted in decreased agricultural production and increased agricultural imports, leading to increased trade deficits and foreign dependency on foreign sources, both of which can contribute to making basic products difficult to acquire [3].

Mexico is not an exception to Latin American trends regarding its agricultural sector. The modernization of farming practices and the use of technology in Mexico’s fields has been at the center of great debate. Because most of Mexico’s land is not suitable for traditional agriculture, modernization of agricultural practices and the principle of competitive advantage infer a transition, in part, to non-traditional crops cultivated with emerging technologies, including wireless technologies, such as sensors and actuators. This automation may not only significantly improve production and crop quality, but more efficiently use often scarce natural resources such as soil and water.

Precision agriculture techniques, whose objective is to efficiently use consumables such as fertilizers, pesticides, soil, and water, among others, can be applied in both open and closed spaces. An additional advantage of precision agriculture techniques is that they can reduce the use of dangerous agricultural products that contaminate the environment. Precision agriculture traditionally involves global positioning (GPS) to help identify problems related to ground monitoring, insect pests, humidity and crop density, among others. Present satellite technology and image analysis, however, can be rather costly and imprecise, as many problems are too small to be detected by satellite imagery.

Sustainable agricultural practices emphasize the development of biotechnology, techniques to increase crop production, and the application of technology to agricultural production, among others. However, these practices make applying state-of-the-art technology in many parts of the world difficult to achieve. Expanding the role of technology to monitor and control crops and otherwise automate agricultural practices is essential to decrease costs and provide benefits to a greater percentage of producers worldwide. Precision agriculture, comprised of sensors, wireless networks, computer hardware, software applications, and wireless communication technologies can significantly reduce the time a producer requires to make important decisions related to resource management, planning, administration, process analysis and evaluation, which ultimately contribute to improved decision making.

Presently, the agricultural sector does not sufficiently employ technology and informatics to modify production practices. Although progress is being made with regards to the deployment of sensors, wireless networks, actuators and other electromechanical devices in agricultural settings, there are still important areas of development that have not been sufficiently explored. The digital divide also affects agricultural practices in developing countries, as many current innovations have not yet “filtered down”. Embedded systems and wireless technologies can, in the long run, reduce costs and increase profits in countries with favorable year-round climates that permit multiple harvests but lack other essentials required to maximize their potential.

Advances in micro-electro-mechanical systems (MEMS) technology have made the deployment of wireless sensor nodes a reality, in part, because they are small, inexpensive and energy efficient. Each node of a sensor network consists of three basic subsystems: a sensor subsystem to monitor local environmental parameters, a processing subsystem to provide computational support to the node, and a communication subsystem to provide wireless communications to exchange information with neighboring nodes. Because individual sensor nodes can only cover a relatively limited area, they need to be connected to one another in a coordinated manner to form a wireless sensor network (WSN), which can provide large amounts of detailed information about a given geographic area. Consequently, a wireless sensor network can be described as a collection of intercommunicated wireless sensor nodes which coordinate to perform a specific action. Unlike traditional wireless networks, WSNs depend on dense deployment and coordination to carry out their task. Wireless sensor nodes measure conditions in the environment surrounding them and then transform these measurements into signals that can be processed to reveal specific information about phenomena located within the coverage area surrounding these sensor nodes.

However, the imperative necessity to control physical variables such as temperature, relative humidity, soil moisture, *etc.*, has led to the development of wireless sensor and actor networks (WSANs), which are commonly composed of heterogeneous devices referred to as sensors and actuators. Sensors are low-cost low-power multi-functional devices that communicate wirelessly for short distances. Actuators are usually resource-rich devices with greater processing capabilities, higher transmission capabilities, and longer battery life. Actuators collect and process sensor data and perform specific actions within a specified environment based on the information they receive.

Future applications will extensively employ wireless sensor networks that function in real time in conjunction with communications systems, mechanical actuators, and even robots to monitor and intervene in crop cultivation. A wireless sensors network (WSN) permits remote monitoring of many parameters, depending on the type of sensors used and the coverage area. This type of network consists of a large number of sensor nodes that are wirelessly connected to each other, to electromechanical devices, and to a communications network, all of which form a triad to monitor and control crop development. Generally, each node of a WSN consists of sensors and/or actuators. Sensors are characterized by their limited memory and computation capacities, but one advantage of sensors is that they require little power to perform their functions. Wireless sensor networks consisting of many nodes are currently being used in densely populated large scale areas. WSNs can have homogenous structures, where all nodes present similar characteristics, or heterogeneous structures, where some nodes are more powerful than others or are differentiated by physical characteristics, including the type of battery or antenna the individual nodes use, or whether specific nodes are static or dynamic.

WSNs have a variety of applications. Examples include environmental monitoring—which involves monitoring air, soil and water, condition-based maintenance, habitat monitoring, seismic detection, military surveillance, inventory tracking, smart spaces, *etc*. [4,5]. Despite their many diverse applications, WSNs pose a number of unique technical challenges because of fault tolerance (robustness), scalability, production costs, operating environment, sensor network topology, hardware constraints, transmission media and power consumption.

In this work, we selected a 100-meter row of watermelons in a 6-hectare field, where we placed sensors linearly at 5-meter intervals. Watermelons were chosen as the crop for study because they require a specific temperature and humidity to optimally ripen. However, our proposed technological platform can also be applied in other precision agriculture applications because it incorporates a modified version of LORA-CBF, a wireless location-based routing algorithm that uses cluster-based flooding, which, as mentioned previously, addresses the scalability concerns of wireless sensor networks, including monitoring of residual battery energy.

The remainder of this paper is organized as follows: Section 2 classifies routing algorithms and their application in wireless sensor networks. Section 3 describes the technological platform for wireless sensor nodes. Section 4 reviews the proposed hierarchical and flat algorithms for our wireless sensor network. Section 5 explains the system evaluation. Section 6 describes the evaluated scenario and the results obtained from the small-scale network and Section 7 summarizes our work and proposes future research.

## 2. Routing Algorithms for Wireless Sensor Networks

Routing protocols for wireless sensor networks can be classified as data-centric, hierarchical or location-based.

### 2.1. Data-Centric Protocols (Flat Architecture)

In data-centric protocols, the sensor nodes broadcast an advertisement for the available data and wait for a request from an interested sink. Flooding is a simple technique that can be used to broadcast information in wireless sensor networks. However, it requires significant resources because each node receiving a message must rebroadcast it, unless a maximum number of hops for the packet are reached, or the destination of the packet is the node itself. Flooding is a reactive technique that does not require costly topology maintenance or complex route discovery algorithms. However, it does have several additional deficiencies, including implosion, overlap and resource blindness [6]. A derivation of flooding is gossiping, in which nodes do not broadcast. Instead, they send the incoming packets to a randomly selected neighbor.

Sensor protocols for information via negotiation (SPIN) address the deficiencies of classic flooding by providing negotiation and resource adaptation [7]. However, the SPIN data advertisement mechanism cannot, by itself, guarantee data delivery [8]. SPIN employs a shortest path strategy based on three types of messages to communicate:

ADV—new data advertisement. When a SPIN node has data to share, it can advertise this fact by transmitting an ADV message containing meta-data.
REQ—request for data. A SPIN node sends a REQ message when it wishes to receive some actual data.
DATA—data message. A DATA messages contains actual sensor data with a meta-data header.

Unlike traditional networks, a sensor node does not necessarily require an identity (e.g., an address). Instead, applications focus on the different data generated by the sensors. Because data is identified by its attributes, applications request data matching certain attribute values. One of the most popular algorithms for data-centric protocols is direct diffusion, which bases its routing strategy on a shortest path strategy [9]. A sensor network based on direct diffusion exhibits the following properties: each sensor node names the data it generates with one or more attributes, other nodes may express interest based on these attributes, and network nodes propagate interests. Interests establish gradients that direct the diffusion of data. In its simple form, a gradient is a scalar quantity. Negative gradients inhibit the distribution of data along a particular path, and positive gradients encourage the transmission of data along the path.

The Energy-Aware Routing protocol is a destination-initiated reactive protocol that increases network lifetime by using a single path at all times, which is very similar to source routing [10]. Rumor routing [11] is a variation of direct diffusion that is mainly intended for applications where geographic routing is not feasible. Gradient-based routing is another variant of direct diffusion [12]. The key idea of gradient-based routing is to memorize the number of hops when the interest is diffused throughout the network. Constraint Anisotropic Diffusion Routing (CADR) is a general form of direct diffusion [13] and lastly, Active Query Forwarding in Sensor Networks (ACQUIRE) [14] views the network as a distributed database, where complex queries can be further divided into several sub queries.

The XMesh Routing Protocol is a multi-hop routing protocol developed by Crossbow to run on the MICA and eKo families of motes [15]. In the XMesh routing protocol the cost metric minimizes the total number of transmissions required to deliver a packet over multiple hops to a destination and is termed Minimum Transmission (MT) cost metric. This differs from the traditional cost metric of distance vector routing which is hop count. The multi-hop network is initially formed when motes broadcast periodic beacon messages to all other motes within radio range. When the beacon messages are sent, they contain a cost value, which indicates the energy required to transmit a message from the motes to the base station. Higher cost indicates that more energy is required to transmit.

### 2.2. Hierarchical Protocols

Hierarchical protocols are based on clusters because clusters contribute to more scalable behavior as the number of nodes increases. Furthermore, clusters provide improved robustness and facilitate more efficient resource utilization for many distributed sensor coordination tasks.

Low-Energy Adaptive Clustering Hierarchy (LEACH) is a cluster-based protocol that minimizes energy dissipation in sensor networks by randomly selecting sensor nodes as cluster-heads [16]. Power-Efficient Gathering in Sensor Information System (PEGASIS) [17] is a near optimal chain-based protocol. The basic idea of the protocol is to extend network lifetime by allowing nodes to communicate exclusively with their closest neighbors, employing a turn-taking strategy to communicate with the Base Station (BS). Threshold-sensitive Energy Efficient protocol (TEEN) [18] and Adaptive Periodic TEEN (APTEEN) [19] have also been proposed for time-critical applications. In TEEN, sensor nodes continuously sense the medium, but data transmission is done less frequently. APTEEN, on the other hand, is a hybrid protocol that changes the periodicity or threshold values used in the TEEN protocol, according to user needs and the application type.

### 2.3. Location-Based Protocols

Location-based protocols make use of position information to relay data to the desired regions, instead of the entire network. Before a packet can be sent, the position of the destination must first be determined. Typically, a location service is responsible for this task. Existing location services can be classified according to how many nodes host the service. This can be either a specific node or all of the network nodes. Furthermore, each location server may maintain the position of a specific node or all the nodes in the network.

In position-based routing, a node’s forwarding decision is primarily based on the position of a packet’s destination and the position of its immediate one-hop neighbor. The position of the destination is contained in the header of the packet. If a node has a more accurate position of the destination, it may choose to update the position in the packet before forwarding it. The position of the neighbors is typically learned through a one-hop broadcast beacon. These beacons are sent periodically by all nodes and contain the position of the sending node.

We can distinguish three main packet-forwarding strategies for position-based routing: greedy forwarding, restricted directional flooding, and hierarchical approaches. For the first two, a node forwards a given packet to one (greedy forwarding) or more (restricted directional flooding) one-hop neighbors that are located closer to the destination than the forwarding node itself. The selection of the neighbor in the greedy case depends on the optimization criteria of the algorithm. The third forwarding strategy is to form a hierarchy in order to scale to a large number of mobile nodes.

Minimum Energy Communication Network (MECN) [20] establishes and maintains a minimum energy network for wireless networks by utilizing low-power geographic positioning system (GPS). The main idea of MECN is to find the sub-network with the smallest number of nodes that requires the least transmission power between any two particular nodes (shortest path). The Small Minimum Energy Communication Network (SMECN) [21] is an extension of MECN. The major drawback of MECN is that it assumes that every node can transmit to every other node, which is not always possible. One advantage of SMECN is that it considers obstacles between pairs of nodes. Geographic Adaptive Fidelity (GAF) [22] is an energy-aware location-based routing algorithm primarily designed for ad-hoc networks that can also be applied to sensor networks. GAF conserves energy by turning off unnecessary nodes in the network without affecting the level of routing fidelity. Finally, Geographic and Energy Aware Routing [23] uses energy-awareness and geographically informed neighbor selection heuristics to route a packet toward the destination region.

## 3. Technological Platform for the Wireless Sensor Node

The design of the agricultural platform system faced three significant challenges: building a sufficiently lightweight, energy efficient hardware capable of monitoring and control physical variables; incorporating and evaluating different operating systems and algorithms into a software to achieve autonomous transfer sensing and control variables; and integrating subsystems such as microprocessor, sensors and actuator modules and wireless networking into a fully functional wireless platform solution.

### 3.1. ARM System and Wireless Radio Networking

**Figure 1.**
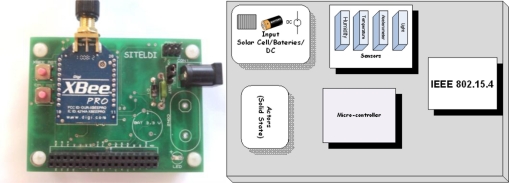
Picture and the block diagram of the wireless sensor node.

This section provides an overview of the ARM microcontroller systems, focusing on the LPC2148F model of the LPC2000 family. Table 1 provides a summary of the LPC2148F hardware and a picture and the block diagram of the wireless sensor node is shown in Figure 1.

The LPC2148F model has an ARM7TDMI-S [24] processor of the ARM architecture, where the S means that it has a synthesized VHDL core. For a more in depth description of the LPC2148F system, the following references should be consulted [25–27]. The agricultural platform is equipped with 802.15.4 compliant radios, namely the XBEE Pro Zigbee radios from Maxstream. These radios were chosen due to their combination of lightweight, long transmission range, serial interface compatibility with the ARM processor, and packet interface. As shown in Figure 1, the small size of the agricultural platform designed at the SITELDI Solutions Laboratory possesses the advantages of being inexpensive, energy efficient and highly resistant in outdoor environments.

### 3.2. Lightweight PaRTiKle Operating System Design

**Figure 2.**
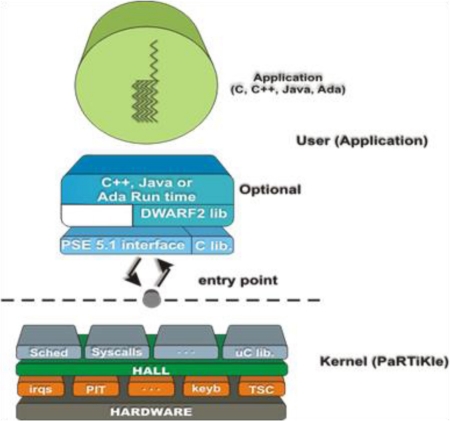
PaRTiKle OS Architecture.

In this section, we describe the architecture of the PaRTiKle operating system, which adheres to a classical layered multi threaded design, as shown in Figure 2.

PaRTiKle [28,29] is a new embedded real-time operating system designed to be as compatible with the POSIX.51 standard as possible. The native API consists of “C” POSIX threads and provides support for C++, Ada and Java (tasking, synchronization, protected objects, exception handling, *etc*.). PaRTiKle has been designed to support applications with real-time requirements, providing features such as full preemptability, minimal interrupt latencies, and all the necessary synchronization primitives, scheduling policies, and interrupt handling mechanisms needed for these types of applications. To meet the application requirements of sensor networks, the PaRTiKle OS implements a lightweight and energy-efficient scheduler, a user-level network stack, as well as other components such as device drivers all this in less than 12 Kbytes code lines. A layered network stack and hardware driver system is included to simplifying communication in an embedded platform. The PaRTiKle OS itself is coded mostly in C, and it presents a simplified C POSIX.51 standard programming interface. An application developer may write the application code in standard ANSI C and compile it with gcc, avoiding the need to learn a specialized language or compiler. PaRTikle’s structure provides several advantages over existing sensor network systems because it:

- is portable, configurable and maintainable
- supports multiple execution environments. This allows it to execute the same application code (without any modification) under different environments. Presently, it can be used in a bare machine and to facilitate application development. It also runs as a Linux regular process and as a hypervisor domain, increasing the number of devices that can participate in a PaRTiKle network.
- supports multiple programming languages. PaRTiKle currently supports Ada, C, C++, and Java (the current support of this last language is only supported when the 4.2 GCC compiler is used).
- supports a great deal of existing code. A significant amount of open-source code can be ported to PaRTiKle OS.

#### 3.2.1. PaRTiKle Architecture

Figure 2 sketches the PaRTiKle architecture. Contrary to other small embedded RTOS, which are implemented as a library that is linked to the application, PaRTiKle has been designed as a real kernel with a clean and well-defined separation between the kernel and application execution spaces. All kernel services are provided via a single entry_point, which improves the robustness and simplifies porting PaRTiKle to other architectures and environments.

#### 3.2.2. Execution Environments

PaRTiKle has been designed to run under several different execution environments. There are presently three different execution environments are available, all of them for the ARM and x86 architecture: (1) on a bare machine, (2) as a Linux regular process and (3) as a domain of XtratuM [30,31]. This last alternative provides the possibility of executing PaRTiKle jointly with another general purpose operating system (Linux so far).

- On a bare machine: PaRTiKle is the only system executed and is in charge of managing all of the hardware. This environment is the best option for applications with real-time constraints.
- As a Linux regular process: This environment is intended for testing purposes and the generated code is executed as a regular Linux process. PaRTiKle still has direct access to the hardware; however, real-time constraints are not guaranteed.
- As an XtratuM domain: XtratuM is a hypervisor that provides hardware virtualization and enables the execution of several kernels (or run-times) concurrently. PaRTiKle can be built to be XtratuM aware and then loaded using the XtratuM.

## 4. Proposed Routing Algorithm for Wireless Sensor Networks

Location Routing Algorithm with Cluster-Based Flooding (LORA_CBF) [32] was developed by the principal author of this work, and was modified to meet the requirements of precision agriculture applications.

LORA-CBF is formed with one cluster-head, zero or more members in every cluster and one or more gateways to communicate with other cluster-heads. Each cluster-head maintains a “Cluster Table.” A “Cluster Table” is defined as a table that contains the addresses and status of neighbor nodes. A node in LORA-CBF can be in any of the following four states:

- Undecided: A node is in this transitional state when it is in search of a cluster-head. Nodes are initially undecided when they enter the network or when they wake up.
- Member: A node that is a member of any cluster assigned to a cluster-head. A member in LORA-CBF cannot retransmit a search packet.
- Cluster-head: A node that is responsible for all the nodes in its cluster. The cluster-head is responsible for periodically transmitting Hello messages. The cluster-head also maintains the cluster table of the member and gateway nodes in its cluster.
- Gateway: A node that is member of at least two cluster-heads that can be used for communication between clusters.

### 4.1. Cluster Formation

To enable cluster formation and maintenance, all nodes keep the information about their neighbors in their neighbor table. Let t be the period of time between the Hello broadcasts. When a node first switches on, it first listens to Hello packets on the broadcast channel. If any other node on the broadcast channel is already advertising itself as a cluster-head (status of node = cluster-head), the new node saves the heard cluster-head ID in its cluster-head ID field and changes its status to member. At any point in time, a node in the mobile network associates itself with a cluster-head. The cluster-heads are identified by the cluster-head ID. Otherwise, the new node becomes cluster-head. The cluster-head is responsible for the cluster and periodically sends a Hello Message.

***Strategy for Cluster-head forwarding***

If (Packet_received)

    If (type_packet == “d”)

        If (know_path_sink) Send_packet_r()

        else Send_packet_s()

    else if (type_packet == “r”)

        if (idpacket == myid)

            If (know_sink) Send_packet_sink()

            else Relay_packet_r()

     else if (type_packet == “t”)

        if (idpacket == myid) Do_task()

        else Relay_packet_t()

    else if (type_packet == “s”)

        if(idpacket == myid)

             if(know_sink) Send_packet_sink()

             else Relay_packet_s()

    else if (type_packet == “z”)

        register_address_sink()

else Lora_cbf()

When a cluster member receives a Hello message, it registers the cluster-head and responds with a reply Hello message. The cluster-head then updates the Cluster Table with the address and status of every member in the cluster. When a member receives a Hello packet from a different cluster-head, it first registers the cluster-head, but the member does not modify its cluster-head ID until the expiration time for the field has expired. Before the member rebroadcasts the new information, it changes its status to a gateway. After receiving the Hello packet, the cluster-heads update the Cluster Table with the information about the new gateway.

***Strategy for Gateway forwarding***

If (Packet_received)

    if (type_packet == “r”)

         if (idpacket == myid)

              If (know_sink) Send_packet_sink()

              else Relay_packet_r()

    else if (type_packet == “t”)

         if (idpacket == myid) Do_task()

         else Relay_packet_t()

    else if (type_packet == “s”)

         if (idpacket == myid)

              if (know_sink) Send_packet_sink()

              else Relay_packet_s()

else Lora_cbf()

If the cluster-head source wants to send a message to the sink, it first checks its routing table to determine if it has a “fresh” route to the sink. If it does, it first seeks its Cluster Table to determine the closest neighbor to the sink. Otherwise, it starts the discovery process.

### 4.2. Routing Strategy for Hierarchical Architecture

**Figure 3.**
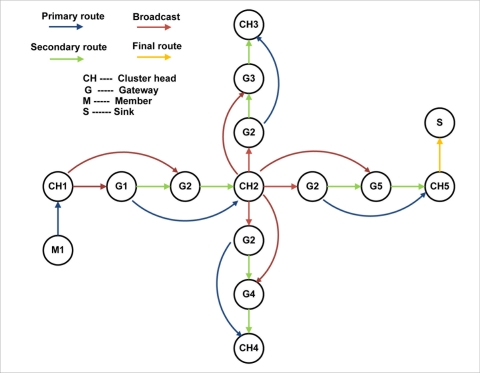
Routing Strategy for hierarchical architecture.

When a node wants to send a packet to the sink, it sends a packet “d” (discovery) to its cluster head (Figure 3).

The cluster head source seeks the route in its routing table. If it has the route, the cluster head source sends a packet “r” (route) to the sink. Otherwise, the cluster head source sends a package “s” (search) to search for a route to the sink. After this, the sink receives an “s” package and it replies to the cluster head source by sending a “t” (target) packet that leads to the cluster head source as the target that generated the “s” package. This package contains the path to the sink. However, if the route to the sink becomes invalid, the cluster head source generates an “f” (fail) package indicating that it was not possible to reach the sink, and rebuilds a package “s” to find a new route to the sink.

### 4.3. Routing Strategy for Flat Architecture

**Figure 4.**
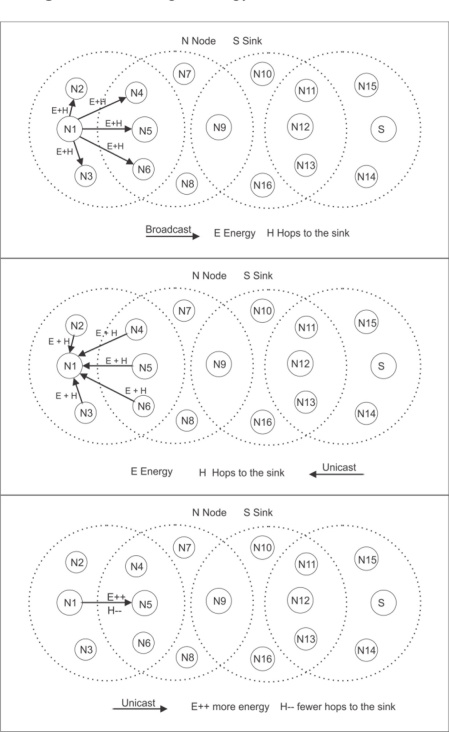
Routing Strategy for flat architecture.

When a node has data to transmit to the sink, it sends a broadcast message to the nodes that are within its coverage area to create a temporary table with the energy and number of hops needed to reach the sink. In the Figure 4, the node possessing the most energy and the requiring the smallest number of hops to the sink is chosen to send the data packet. This process is repeated until the data packet reaches the sink. If a data packet fails to reach the sink, this process is repeated, beginning at the source, until it successfully reaches the sink.

## 5. System Evaluation

The following metrics were used to evaluate the LORA-CBF algorithm in a hierarchical and a flat architecture.

- Route discovery time (latency): the amount of time the source has to wait before sending the first data packet.
- Packet delivery ratio: the ratio of the number of data packets delivered to the destination and the number of data packets sent by the sender. Data packets may be dropped en route exclusively if the next hop link is broken at the moment the data packet is ready to be transmitted.
- Average end-to-end delay of data packets: all of the possible delays caused by buffering during route discovery, queuing at the interface queue, re-transmission delays at the MAC, and propagation and transfer times.
- Throughput: the average rate of successful message delivery over a communication channel.
- Routing load: the routing packets transmitted per data packet transmitted. This provides an idea of network bandwidth consumed by routing packets with respect to ‘useful’ data packets.
- Overhead (packets): the total number of routing packets that are generated divided by the total number of data packets transmitted, plus the number total routing packets.

**Figure 5.**
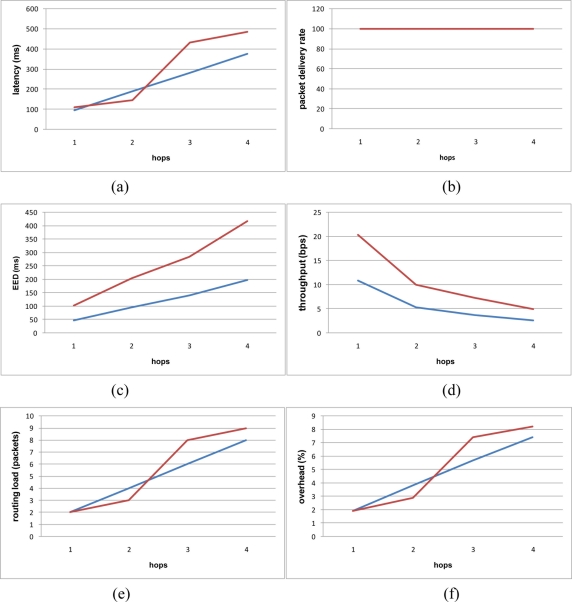
**(a)** Route Discovery Time; **(b)** Packet Delivery Ratio; **(c)** End-to-End Delay; **(d)** Throughput; **(e)** Routing Load; **(f)** Overhead.

In the Figure 5, the red line represents the hierarchical architecture and the blue line the flat architecture. Figure 5(a) shows that the latency of the flat algorithm increases linearly in proportion to the number of hops. On the other hand, the latency of the hierarchical algorithm increases irregularly because of its group formation mechanism. However, it is important to note that regardless of whether the algorithm is flat or hierarchical, latency increases proportionally to the number of hops. Figure 5(b) shows the Packet delivery ratio. In this scenario, both the flat and hierarchical algorithms behave the same. Figure 5(c) shows that End-to-End delay increases proportionally for both algorithms, although the flat algorithm’s End-to-End delay increases linearly while the hierarchical algorithm’s increase in End-to-End delay is more irregular due to its group formation mechanism. This mechanism substantially increases End-to-End delay. Figure 5(d) represents the throughput which is significantly better in the hierarchical algorithm because of its superior packet transmission mechanism, which substantially reduces the possibility of collisions. Figure 5(e) shows the routing load. The flat algorithm performs better than the hierarchical algorithm with regards routing load because it does not have a group formation mechanism that can increase packet transmission. Figure 5(f) shows overhead (packets) which is the total number of routing packets that are generated divided by the total number of data packets transmitted, plus the number total routing packets. Again, the flat routing algorithm performs better than the hierarchical algorithm with regards to overhead.

In summary, the hierarchical architecture adds more delay in terms of route discovery time, End-to-End delay, routing load and overhead, but it significantly improves throughput. The main advantage of using the hierarchical architecture *vs*. the flat architecture is network scalability. The hierarchical architecture is more scalable and can handle a greater number of nodes.

## 6. Evaluated Scenario

**Figure 6.**
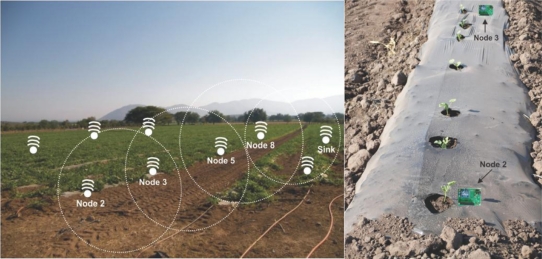
Scenario evaluated.

The scenario in Figure 6 was used to evaluate the proposed routing algorithms. Five wireless sensor nodes were employed to represent a small-scale network. Node N2 represents the node which starts the routing process.

The wireless sensor network consisted of 20 sensors that were placed linearly at 5 meter intervals. The position of the four wireless sensor nodes is shown in Figure 6. The temperature and humidity sensors were placed on wooden rods placed in the soil. We chose to place the nodes in this way because we wanted the average air temperature and humidity at the intermediate height between the plants. The soil moisture and temperature sensors were placed approximately 5 cm from each other at each 5-meter interval. The sensors were then placed just in the soil a few millimeters below the actual ground level.

Each wireless sensor node has four ports, so we connected three soil moisture and temperature sensors and one humidity and temperature sensor per wireless sensor node sensing each one of the variables every hour. The wireless sensor network was deployed on Monday 13/12/2010 at 13:00, approximately, and was recovered on Wednesday 15/12/2010 at 12:00.

### Results Obtained from the Small-Scale Network

**Figure 7.**
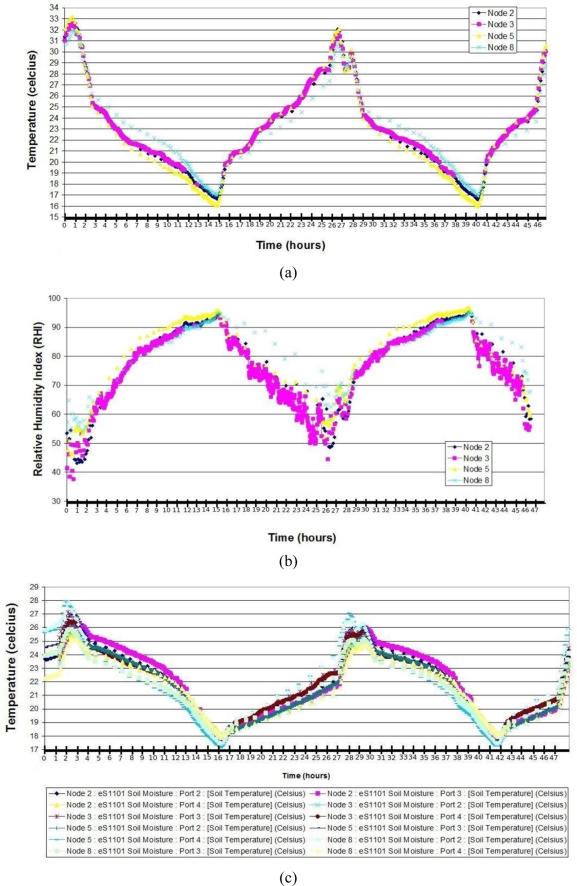

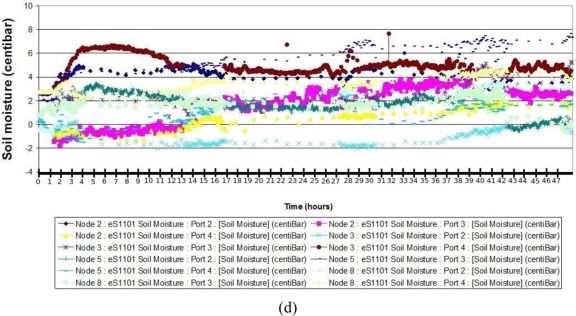
**(a)** Ambient temperature; **(b)** Relative Humidity; **(c)** Soil temperature; **(d)** Soil moisture.

Figure 7(a) shows the ambient temperature in degrees Celsius. The wireless sensor network was deployed at 13:00 on Monday in a field as shown in Figure 6. On Monday, the temperature reached 33 °C and on Tuesday, the temperature reached 32 °C between 13:00 and 15:00 P. M. The primary difference between the two days was the greater cloud density on Tuesday. However, the minimum temperature was the same on both days reaching a nightly low of 16 °C. Figure 7(b) provides the relative humidity experienced on both days. The maximum relative humidity reached was 95 (Relative Humidity Index, RHI), which is a typical outdoor field environment in this region. The minimum relative humidity was 40 RHI on Monday, which increased to 50 RHI on Tuesday because of the increased cloudiness that day. This difference, however, allows us to infer that RHI increases with exposure to sunlight. Figure 7(c) shows the soil temperature. Similarly to the ambient temperature, the soil temperatures were slightly affected by the cloud density on Monday. The maximum temperature of 28 °C was reached between 13:00 and 15:00 hours on Monday and a temperature of 27 °C between 14:00 and 16:00 on Tuesday. The minimum temperature was 17 °C for both days. Figure 7(d) presents soil moisture results. The soil moisture results differ significantly from plant to plant because the plants are irrigated manually using traditional methods. Consequently, the water is not distributed uniformly among the plants and causes different soil moistures, depending on the irrigation time and actual water flow each plant receives.

## 7. Conclusions

In this work, a new platform for wireless sensor networks including its embedded operating system and its routing algorithm was evaluated in terms of route discovery time, packet delivery ratio, End-to-End delay, Throughput, routing load and overhead. A flat and hierarchical algorithm was evaluated in a small-scale network under test bed conditions in a watermelon field. The flat algorithm proved to be superior with regards to route discovery time, End-to-End delay, and routing load and overhead. The hierarchical algorithm proved to be superior regarding Throughput and scalability. In small-scale network applications, we found that the flat algorithm is more suitable because of its simplicity. Results show that LORA_CBF is suitable for both flat and hierarchical algorithms and is suitable for small-scale agricultural use. We conclude that our proposed technological platform with a modified version of the LORA_CBF routing algorithm can also be applied to precision agriculture because it is a wireless location-based routing algorithm that uses cluster-based flooding and monitors residual battery energy.

## Figures and Tables

**Table 1.** Summary of LPC2148F specifications.

**Model**	**LPC2148F**
Processor	ARM7TDMI-S 60 MHz
RAM memory	32 Kbyte (expandable module external)
ROM memory	512 Kbyte EEPROM (electrically-erasable programmable ROM)
Serial ports	UART: serial (38,400 bauds) + UART1: modem
Clocks	RTC 32.768 KHz
Timers	T0, t1: 15 MHz (CCLK = 60 MHz/PDIV = 4)
others	Gpio, spi, pwm, i2c, and can
